# Last Illness of Valentine Mott, M. D.

**Published:** 1866-03

**Authors:** 


### SELECTED ARTICLES.

### *Last Illness of Valentine Mott, M. D.*

The following account of the last illness of Valentine Mott, M. D., presented by Dr. Austin Flint to the N. Y. Academy of Medicine, will be read with interest:

MR. PRESIDENT,—In compliance with a request made at the Special Meeting of the Academy, held on the occasion of the death of the late Valentine Mott, I have the honor to submit a succinct report of his last illness. In making this report, I shall not presume to encroach upon the duty and privilege, belonging more appropriately to others, of rehearsing the surgical achievements which will render memorable in all time the name of Valentine Mott; or of pronouncing eulogiums for private virtues, which made him not less worthy of admiration as a man than as an illustrious member of our profession.

For several months preceding his death, the family and friends of Dr. Mott had observed a manifest decline in his physical vigor. He had pain (supposed to be neuralgic) in the back and limbs. The pain was at times exceedingly severe. Under a belief that the neuralgia was due to malaria, he took, for a time, arsenic. Cupping, with and without scarification, was also resorted to. I am unable to give further details with respect to these and other measures of treatment prior to his last illness. There was no evidence of failure as regards his mental faculties. A short time only before his last illness, I was associated with him in an important case, in which, from his intimate relations with the patient, he had consented to assume the responsibility of the attending physician. In the management of this case, the accuracy and completeness with which he carried in his mind the surgical events, and his attention to minute therapeutical details, impressed me strongly. The recent tragic events which producod such a profound sensation throughout our country—I refer to the assassination of our late President—occurred a few days prior to his last illness. He was very deeply affected by this event; so much so, that the members of his family entertain a conviction that it contributed to his illness. His utterances in delirium frequently related to circumstances connected with this event. It is to be added, that he had repeatedly had attacks of intermitting fever.

On Saturday, April 22d, without any unusual exertion, exposure, or apparent exciting cause of any kind, he was seized with a severe chill at two o'clock P. M. The chill lasted an hour. It was accompanied with great prostration and intense lumbar pain. During its continuance he was seen by Dr. Vanderpoel. It was followed by intense febrile movement. I saw him at 5½ o'clock P. M. At this time the skin was extremely hot, the pulse frequent, the intense lumbar pain continued, and he was greatly prostrated. He was then lying upon a sofa in his office. It was necessary to carry him up stairs to his bed-chamber. This was done, and a quarter of a grain of the sulphate of morphine given. At 7 P. M. he began to perspire. At 10½ P. M. he had perspired freely, and the febrile movement had ceased. He appeared to have passed through an unusually severe paroxysm of intermitting fever. The lumbar pain did not continue, but he now, for the first time, complained of intense pain in the left leg, the pain being referred especially to the calf and ankle. A suppository containing half a grain of the morphine was given.

*April* 23. I was requested to see him at 6 A. M. He had passed a wretched night, from the continuance of intense pain in the left leg. A quarter of a grain of the sulphate of morphine was given by the mouth. Relief of the pain followed, and, during this day, he slept much of the time in short naps. Fearful of a repetition of the paroxysm of fever, two grains of the sulphate of quinine were given hourly, from 11 A. M. to 5 P. M., and, during the night, the same doses were continued at intervals of three hours. During this day he was free from febrile movement. At evening he seemed quite comfortable. He had taken during the day, beef tea, chicken soup, and wine whey, pretty freely. At this time the left leg presented no morbid appearance, but there was notable tenderness over the calf and ankle.

*April* 24. He had passed a comfortable night, but he suffered great pain in the left leg whenever it was moved. He appeared to doze most of the day, but complained of getting no sleep. Swelling and redness of the ankle and foot were observed on this day, and hardness over the calf of the leg. The heat of the limb was considerably increased. At evening some vesication had occurred over both ankles. The pulse on this day became quite irregular and feeble. He had some deafness and tinnitus, attributable to the quinine. He was disinclined to take nourishment, and at times complained of nausea. No dejection had occurred since the attack. The urine was abundant. Auscultation of the heart revealed no murmur. The heart-sounds were feeble, especially the first sound. The apex-beat was not appreciable; but, judging of its situation by the maximum of intensity of the first sound, and determining the area of præcordial dullness by percussion, there appeared to be no enlargement of the heart. During this day the sulphate of quinine was continued in two-grain doses, repeated every two hours until evening. There was no recurrence of the paroxysms of fever. A little wine and brandy, with nourishment, were given on this day. He was reluctant to take either food or stimulants. In the afternoon an eighth of a grain of the sulphate of morphine was given. There was considerable tremulousness of the muscles of the jaw, and of the hands whenever he made any voluntary movements. Some incoherency was observed on this day. During this day, as on the previous day, he was seen repeatedly by Dr. Vanderpoel and myself, together and in alternation.

*April* 25. He had passed a pretty comfortable night, sleeping, at best, half of the night. Brandy and nourishment had been given sparingly, on account of his great repugnance to both. The pulse continued quite irregular. The tongue on this day became dry. The skin was warm. The left leg presented increase of swelling over the ankle and foot, and of hardness over the calf. The blisters over the ankles had enlarged, and presented a dark or purplish appearance. He had a free dejection, the first since the attack. There was great tremulousness of the hands with voluntary movements. Frequent tremulousuess of the jaw continued. He still took food and stimulants very reluctantly. During this day there was considerable incoherency. The urine examined on this day showed a trace of albumen. Its reaction was acid; sp. gr. 1022: urates were deposited in abundance. With the microscope nothing was discovered in the sediment but amorphous urates.

Up to this date only Dr. Vanderpoel and myself were associated in attendance. Dr. Alexander B. Mott returned home from the army on this date. On examination of the affected lower limb, Dr. Mott ascertained diminished force of pulsation of the femoral artery, as compared with the pulsation of the corresponding artery of the opposite limb. In the afternoon there was constant delirium, manifested by talking incoherently without cessation, imagining he was away from home, desiring to get up, the delirium being like that in typhus and typhoid fever. An eighth of a grain of the sulphate of morphine was given without any tranquilizing effect, and afterwards a quarter of a grain.

At 8½ P. M. he was seen in consultation by Dr. Alexander H. Stevens and Dr. Joseph M. Smith, and at 10 P. M. by Dr. Metcalfe. The restless, talkative delirium had ceased. He was somnolent, but easily aroused, recognizing persons who addressed him, and replying to questions, but relapsing into somnolency directly after being aroused. The skin was warm and moist; the pulse was frequent, feeble, and irregular. The rythm of the respirations was disturbed, breathing being suspended at intervals for several seconds. The left leg presented, in addition to the appearances already stated, a circumscribed dark patch on the dorsal aspect of the foot. The slightest movement of this limb now, as before, excited expressions of pain. The gentlemen who saw him in consultation with Dr. Vanderpoel and myself, advised only the continuance of alcoholic stimulants and nourishment.

*April* 26. During the greater part of the preceding night I was in attendance. He was in a semi-comatose state; the respirations and pulse were irregular; deglutition was unaffected, but food and stimulants were given with considerable difficulty on account of his repugnance to them. He remained during the day in this condition. The left leg presented increased vesication over the ankles, and enlargement of the dark patch on the dorsal aspect of the foot near the toes. With considerable difficulty food and stimulants were given at short intervals during the day. He became more and more lethargic; the pulse became progressively more feeble and irregular; the temperature of the skin diminished, and it was covered with clammy perspiration; deglutition became difficult, and death took place at 1 P. M.

The duration of the illness was four days and eight hours.

A review of this report, which, I should state, is essentially a transcript of memoranda noted daily during the illness, shows the following order of events:

*First.* A paroxysm, having all the characters of a paroxysm of intermittent fever, the cold stage lasting an hour, the hot stage lasting four hours, and the sweating stage followed by complete asphyxia. The paroxysm severe, and accompanied by great prostration.

*Second.* Severe pain in the left leg, following the febrile paroxysm, the pain accompanied by great tenderness. On the third day, the occurrence of swelling, redness, increased heat and vesication. On the fourth day, increase of vesication, and the appearance of a dark circumscribed patch on the dorsal aspect of the foot. And, on the fifth day, increase of vesication and enlargement of the dark circumscribed patch. In conjunction with these local appearances, diminished force of pulsation in the femoral artery.

*Third.* Great prostration remaining after the febrile paroxysm. On the third day, incoherency; on the fourth day, typhoid delirium; and, on the fifth day, gradually developed coma, continuing and increasing until death—the mode of dying being chiefly by asthenia, notwithstanding the comatose condition and the disturbance of the rythm of respiration.

As regards the nature of the illness, or the pathological conditions involved, the following conclusions were drawn from the events of the clinical history: The primary affection was intermitting fever—a recurrence of the paroxysm being probably prevented by the use of quinine. The affection of the leg tended rapidly to gangrene, and there was reason to suspect the existence of embolism. The typhoid phenomena, occurring after the febrile paroxysm and during the development of the affection of the leg, would render the name *typho-malarial fever* appropriate; considering this name, in this instance, as denoting the existence of the typhoid state, not typhoid fever, with a malarial fever. The explanation of the fatal termination by exhaustion, after so short an illness, is to be found in the severity of the febrile paroxysm and the local affection, taken in connection with the advanced age of the patient, and the recent deterioration of the constitutional vigor.

These pathological views are believed to represent fairly the opinions of those who were associated in consultation; but it is proper to state that I have not been able to submit to them this report before presenting it to the Academy.—*N. Y. Medical Journal*, Aug., 1865.

